# Plasma and cerebrospinal fluid concentrations of neurofilament light protein correlate in patients with idiopathic normal pressure hydrocephalus

**DOI:** 10.1186/s12987-023-00455-y

**Published:** 2023-07-06

**Authors:** A. Jeppsson, Å. Sandelius, A. Zettergren, S. Kern, I. Skoog, K. Blennow, H. Zetterberg, C. Wikkelsø, P. Hellström, M. Tullberg

**Affiliations:** 1 Hydrocephalus Research Unit, Department of Clinical Neuroscience, Institute of Neuroscience and Physiology, The Sahlgrenska Academy, University of Gothenburg, Sahlgrenska University Hospital, Blå Stråket 7, 41345 Gothenburg, Sweden; 2 Clinical Neurochemistry Laboratory, Department of Psychiatry and Neurochemistry, Institute of Neuroscience and Physiology, The Sahlgrenska Academy, University of Gothenburg, Gothenburg, Sweden; 3 Neuropsychiatric Epidemiology Unit, Department of Psychiatry and Neurochemistry, Institute of Neuroscience and Physiology, Sahlgrenska Academy, University of Gothenburg, Mölndal, Sweden; 4 Department of Neurodegenerative Disease, UCL Institute of Neurology, Queen Square, London, UK; 5 UK Dementia Research Institute at UCL, London, UK; 6 Hong Kong Center for Neurodegenerative Diseases, Clear Water Bay, Hong Kong, China; 7 Wisconsin Alzheimer’s Disease Research Center, University of Wisconsin School of Medicine and Public Health, University of Wisconsin-Madison, Madison, WI USA

**Keywords:** Idiopathic normal pressure hydrocephalus, Neurofilament light chain protein, NFL, Biomarkers, CSF, Plasma

## Abstract

**Background:**

Neurofilament light chain protein (NFL), a marker of neuronal axonal degeneration, is increased in cerebrospinal fluid (CSF) of patients with idiopathic normal pressure hydrocephalus (iNPH). Assays for analysis of NFL in plasma are now widely available but plasma NFL has not been reported in iNPH patients. Our aim was to examine plasma NFL in iNPH patients and to evaluate the correlation between plasma and CSF levels, and whether NFL levels are associated with clinical symptoms and outcome after shunt surgery.

**Methods:**

Fifty iNPH patients with median age 73 who had their symptoms assessed with the iNPH scale and plasma and CSF NFL sampled pre- and median 9 months post-operatively. CSF plasma was compared with 50 healthy controls (HC) matched for age and gender. Concentrations of NFL were determined in plasma using an in-house Simoa method and in CSF using a commercially available ELISA method.

**Results:**

Plasma NFL was elevated in patients with iNPH compared to HC (iNPH: 45 (30–64) pg/mL; HC: 33 (26–50) (median; Q1–Q3), p = 0.029). Plasma and CSF NFL concentrations correlated in iNPH patients both pre- and postoperatively (r = 0.67 and 0.72, p < 0.001). We found only weak correlations between plasma or CSF NFL and clinical symptoms and no associations with outcome. A postoperative NFL increase was seen in CSF but not in plasma.

**Conclusions:**

Plasma NFL is increased in iNPH patients and concentrations correlate with CSF NFL implying that plasma NFL can be used to assess evidence of axonal degeneration in iNPH. This finding opens a window for plasma samples to be used in future studies of other biomarkers in iNPH. NFL is probably not a very useful marker of symptomatology or for prediction of outcome in iNPH.

## Introduction

Idiopathic normal pressure hydrocephalus (iNPH) is a treatable neurological disease in the elderly, characterized by gait and balance disturbance, cognitive decline and urinary incontinence in combination with ventriculomegaly [1]. The etiology remains largely unknown. Up to 80% of patients improve after shunt insertion [2, 3]. Accurate pre-operative identification of shunt responders is difficult, where the lack of reliable laboratory tests is one important factor, which probably contributes to the fact that only 20–40% of patients with iNPH are treated [4]. Thus, better methods for diagnosis and prediction of outcome after shunt surgery are needed [2, 5].

Cerebrospinal fluid (CSF) neurofilament light chain protein (NFL_CSF_) is a biomarker reflecting neuronal death and axonal degeneration [6]. However, the pathophysiological specificity for NFL_CSF_ has proven rather unspecific for neurological disorders [7–9]. NFL_CSF_ is elevated in patients with iNPH in comparison to neurologically healthy individuals [7, 10–15] and a higher NFL is associated with more severe symptoms [11, 13] indicating axonal degeneration as part of the iNPH pathophysiology. After shunting, concentrations have been more contradictory and both increases [14], decrements [16] and unchanged levels [11] in comparison to preoperative values have been reported in lumbar CSF.

It is now possible to determine NFL in plasma (NFL_plasma_), allowing monitoring of disease related axonal degeneration without a lumbar puncture [17, 18]. NFL_plasma_ has been tested successfully in other neurological disorders, such as Alzheimer’s disease (AD) [19], parkinsonian disorders [20, 21] and HIV-associated dementia (HAD) [17] and could open the field to a less invasive biomarker sampling for diagnosis and monitoring of disease. A recent meta-analysis determined the pooled correlation coefficient between CSF and blood NFL r = 0.72 but with considerable heterogeneity between studies [22]. As the results varies between disorders, there is a need to investigate the potential use of NFL_plasma_ in patients with iNPH.

### Aim

The aim of this study was (1) to explore if NFL_plasma_ is elevated in patients with iNPH in comparison to healthy individuals; (2) to explore the association between NFL_plasma_ and NFL_CSF_ in iNPH patients pre- and postoperatively; (3) to explore the associations between NFL concentrations in plasma and CSF and severity of symptomatology and outcome after shunt surgery in iNPH.

## Methods

### Study population

Fifty patients (34 men and 16 women) aged 73; 63–78 years (median; Q1–Q3)) consecutively diagnosed were treated with shunt insertion for iNPH according to the international guidelines [23] at the Hydrocephalus Unit, Sahlgrenska University Hospital between 2014 and 2015 were included. All patients were clinically evaluated pre- and 6–9 months postoperatively by an experienced neurologist, a physiotherapist, and a neuropsychologist.

Symptoms and signs were assessed on the iNPH scale [24] comprising gait, balance, cognition and incontinence domains yielding a score of 0–100 points. Improvement was defined as ≥ 5 points increase in iNPH scale score postoperatively [24].

All patients received a Medtronic Strata ventriculoperitoneal shunt with an adjustable valve. All patients were operated on by frontal approach. All shunts were set to 1.5 opening pressure at time for the insertion. No adverse events were recorded. All shunts were checked for patency and were working at the time of follow-up.

Fifty healthy individuals from the H70-study population based sample reported elsewhere (34 men and 16 women aged 73; 71–80 (median; Q1–Q3)) were included as controls for NFL_plasma_ [25–27]. All controls were non-demented individuals, defined as an MMSE of 29 or above. Controls were matched with regard to age and gender. Demographic data at baseline are given in Table 1.

**Table 1:** Demographic data of 50 iNPH patients at baseline and 50 healthy controls (HC)

	iNPH	HC
Sex (m/f)	34/16	34/16ns
Age (median, Q1-Q3)	73 (69–78)	73 (71–80)ns
MMSE (median, Q1-Q3)	25 (21–28)	29 (29–30)***
BMI (median, Q1-Q3)	28 (25–31)	25 (23–27)**
Diabetes (yes/no)	28% (14/35)	10% (5/45)*
Hypertension (yes/no)	60% (30/19)	38% (19/31)*
Symptom duration, months	36 (24–66)	
Time to follow-up, months	9 (8–11)	

MMSE: Mini mental state examination; BMI: Body mass index; *p < 0.05; **p < 0.01; ***p < 0.001

### CSF and plasma sampling in iNPH and healthy controls

Lumbar CSF was obtained from the iNPH patients at the pre-op clinical work-up and at the post-op examination after median 9 months (Q1–Q3 8–11). Lumbar puncture (LP) was performed in the L3/L4 or L4/L5 interspace with the patient in a lateral recumbent position. The CSF opening pressure was measured. The CSF was collected in polypropylene tubes, centrifuged, aliquoted, and analyzed according to standardized procedures.

Blood samples were collected in EDTA tubes from the iNPH patients at the time for LP both pre- and postoperatively and for controls in connection with the clinical study evaluation [27]. Plasma was processed, aliquoted and stored at − 80 °C until analyzed according to standardized procedures.

### NFL quantification in CSF and plasma

The concentration of NFL_CSF_ was measured by the NF-Light ELISA as described by the manufacturer (UmanDiagnostics, Umeå, Sweden).

The concentration of NFL_plasma_ was measured using the in-house Simoa NFL assay which has been described in detail elsewhere [28]. Briefly, paramagnetic carboxylated beads (Quanterix Corp, Boston, MA, USA) was coated with a mouse anti-neurofilament light antibody (UD1, UmanDiagnostics, Umeå, Sweden) and incubated 35 min with sample and a biotinylated mouse anti-neurofilament light antibody (UD2, UmanDiagnostics) in a Simoa HD-1 instrument (Quanterix). The bead-conjugated immunocomplex was thoroughly washed before incubation with streptavidin-conjugated β-galactosidase (Quanterix). After additional washes, resorufin β-d-galactopyranoside (Quanterix) was added and the immunocomplex was applied to a multiwell array designed to enable imaging of every single bead. The average number of enzymes per bead (AEB) of samples was interpolated onto the calibrator curve constructed by AEB measurements on bovine NFL (UmanDiagnostics) serially diluted in assay diluent. The average inter-assay CV was 7.9%CV for QC1 and 13.4%CV for QC2. The limit of detection (LOD), determined as the mean blank signal + 3 SD for the Simoa NFL assay was 0.3 pg/mL, and the lower limit of quantification (LLOQ) determined as the mean blank signal + 10 SD was 2.7 pg/mL when compensated for a four-fold sample dilution.

### Statistics

Non-parametric methods were used for all analyses due to markedly skewed distributions of concentrations in CSF and plasma and because of the ordinal level of measurement used in some of the domains of the iNPH scale. The Chi-square test was used to compare frequencies across groups. When analysing the relation between paired variables, the Related-Samples Wilcoxon Signed Rank Test was used. For unpaired variables, Mann–Whitney U-test was performed. For associations between two independent variables, the Spearman Rank Order Correlation test was used (r_s_). The level of significance chosen was 0.05, if not otherwise stated. No correction for the mass-significance effect was made. Statistical analyses were performed using IBM® SPSS® Statistics for Windows version 24.

## Results

Thirty-six (72%) patients improved after shunt surgery. The total iNPH scale score increased from 51; 37–72 (median; Q1–Q3) to 70; 53–83 (p ≤ 0.001) and increased scores were seen in all domains (Table 2).

**Table 2:** iNPH scale score (domains and total) in 50 iNPH patients, pre- and postoperatively (median, Q1-Q3 range). Outcome = postoperative change in iNPH scale score

	Preoperatively	Postoperatively	Outcome
	n = 50	n = 50	
Gait	42 (35–81)	71 (43–90)	10 (0–26) ***
Balance	67 (50–83)	67 (50–83)	0 (0–17)**
Neuropsychology	50 (23–67)	60 (32–75)	8 (0–18)***
Continence	40 (20–65)	80 (40–100)	20 (0–40)***
Total	51 (38–73)	70 (53–83)	11 (4–22)***

**p ≤ 0.01; ***p ≤ 0.001

**Fig. 1:**
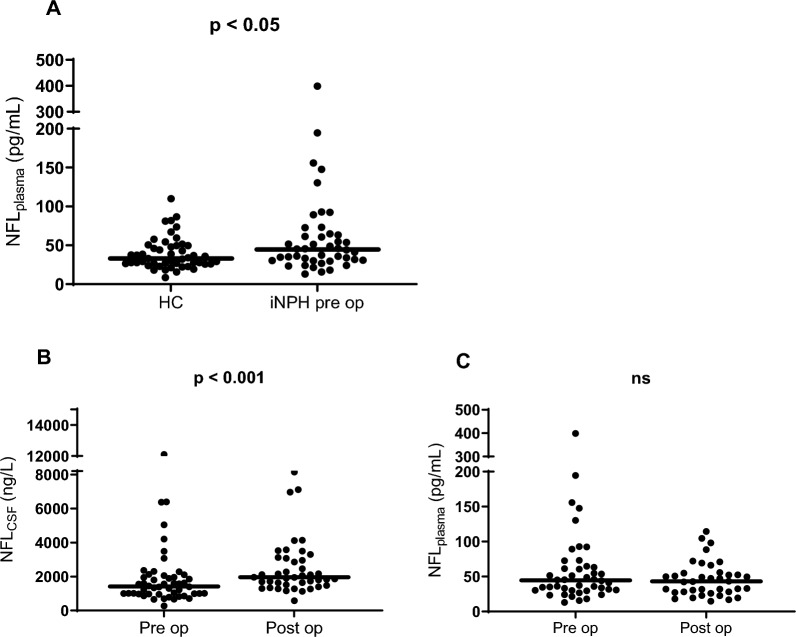
NFL in iNPH patients and healthy controls (HC). **A** NFL_plasma_ in patients with iNPH and healthy individuals (HI), **B** NFL_CSF_ in iNPH pre- and postoperatively, **C** NFL_plasma_ in iNPH patients pre and postoperatively. Line indicating median

Preoperatively, NFL_plasma_ in iNPH patients was 45 (30–64) pg/mL (median; Q1–Q3) compared to 33 (26–50) in controls (p = 0.029) (Fig. 1). NFL_CSF_ was 1415 (985–2063) ng/L in iNPH-patients. Seventeen of the 50 patients (34%) had elevated NFL_CSF_ concentrations compared to the laboratory reference value < 1850 ng/L.

Postoperatively, NFL_plasma_ remained unchanged (43 (28–55) pg/mL, p = 0.54) whereas NFL_CSF_ increased (1955 (1500–2083), p < 0.001). This increase was seen in both patients with normal, or increased, NFL_CSF_ preoperatively.

**Fig. 2:**
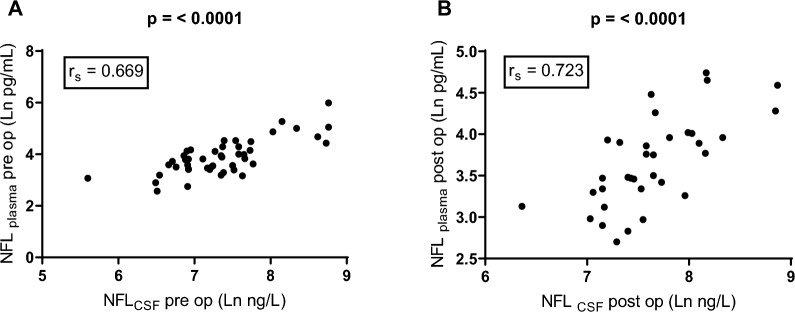
Scatterplot showing correlations between plasma and CSF concentrations of NFL pre- and postoperatively in 50 patients with iNPH. Values are log transformed to reduce skewness. **A** Preoperative, **B** Postoperative.

NFL_plasma_ correlated with NFL_CSF_ both pre- and postoperatively (iNPH patients); r_s_ = 0.629 (p =  < 0.001) and r_s_ = 0.722 (p =  < 0.0001) respectively (Fig. 2).

Preoperatively, a higher NFL_CSF_ correlated weakly with a lower score on the total iNPH scale (r_s_ = − 0.31, p = 0.029) as well as on the domains of gait (r_s_ = − 0.33, p = 0.020), balance (r_s_ = − 0.36, p = 0.010) and neuropsychology (r_s_ = − 0.36, p = 0.012). A higher NFL_plasma_ correlated weakly with a lower score on the total iNPH scale (r_s_ = − 0.33, p = 0.030) as well as on the neuropsychology domain (r_s_ = − 0.35, p = 0.022). Postoperatively, no significant correlations were seen, except between NFL_CSF_ and the balance domain (r_s_ = − 0.39, p = 0.01). Neither did the clinical improvement correlate to concentration of NFL in plasma or in CSF, pre- or postoperatively. Patients who improved after shunt surgery did not differ from those who did not improve in baseline concentrations of NFL_CSF_ or NFL_plasma_ (p = 0.12 and 0.72 respectively).

NFL_CSF_ or NFL _plasma_ was not correlated with age in iNPH patients (r_s_ = 0.28, p = 0.053 and r_s_ = 0.22, p = 0.15 respectively), while there was a weak correlation between NFL_plasma_ and age in controls (r_s_ = 0.39, p = 0.006). There was no correlation between NFL_CSF_ or NFL_plasma_ and disease duration (iNPH patients) (r_s_ = 0.13, p = 0.38 and r_s_ = 0.10 p = 0.38, p = 0.54 respectively).

## Discussion

We report, for the first time, an elevation of plasma NFL in patients with iNPH in comparison with healthy individuals matched for age and gender and that CSF NFL concentrations are correlated with plasma concentrations both pre- and postoperatively. We found only weak correlations between plasma or CSF NFL and measures of clinical symptoms. A postoperative increase of NFL was seen in CSF but not in plasma.

### Elevated NFL in plasma and CSF in iNPH patients

The elevated concentrations of NFL in plasma in comparison with healthy controls reported here corroborate earlier studies using CSF samples [10–16]. Neurofilament protein is the dominant protein of the axonal skeleton that is comprised of three subunits; a light, a medium and a heavy chain that refers to differences in their C-terminus [29]. The light subunit NFL is used as a biomarker for neuroaxonal damage and is elevated in a large number of neurological diseases [9].

Even if most studies report CSF NFL to be elevated in iNPH in comparison to healthy controls, the elevation seen in the clinical setting is normally mild-moderate and according to our experience not necessarily above laboratory reference value. In our material, 34% of the patients had concentrations above this reference value which is in accordance with this notion. In contrast, markedly elevated levels of NFL are often seen in some of the clinical mimics of iNPH, such as atypical parkinsonian disorders, Alzheimer’s disease and frontotemporal dementia [9, 30]. Thus, a marked elevation of NFL in CSF should inform the clinician to consider potential differential diagnoses or comorbidities which might have impact on prognosis and treatment considerations. The mild elevation of NFL in iNPH is consistent with a less aggressive deterioration and might represent a less active destruction of axons in iNPH. This notion is also coherent with the clinical reversibility and the subcortical nature of the disease and is supported by recent resting-state functional MRI findings indicating partially reversible plasticity functional mechanisms in iNPH as well as the postoperative improvement in periventricular white matter perfusion seen in shunt responders [31, 32].

### Correlation of NFL in plasma and CSF

Plasma NFL concentrations were significantly correlated with CSF NFL concentrations both pre- and postoperatively with a strength of associations similar to that in other neurological disorders [22] supporting the notion that plasma concentrations of NFL reflect CSF concentrations also in iNPH. This relationship is not self-evident: the altered CSF dynamics in iNPH as well as the shunt treatment, adding a new drainage route of CSF from the ventricles to the peritoneal cavity, or to the atrium of the heart, can both possibly affect the CSF/plasma protein relationship why this relationship needs to be confirmed in studies like this. The NFL efflux routes and the CSF-plasma clearance mechanisms are largely unknown but a recent report indicates that glymphatic and meningeal lymphatic clearance functions may be involved in both individual- and disease specific manners with daytime variation, suggesting that CSF clearance is more dominant for NFL than for brain Amyloid beta proteins being excreted by different routes [33]. The unchanged magnitude of the correlation between CSF and plasma NFL even after shunt surgery strengthens the clinical value of plasma measurements, and further indicates that the CSF-plasma clearance is preserved after insertion of a shunt and that the direction of absorption of NFL into the blood plays a minor roll. This study supports that NFL, and most probably other biomarker proteins, can be analyzed in blood for diagnostic or prognostic purposes, to monitor disease or to evaluate core pathophysiological mechanisms in iNPH. This also opens a novel field of more non-invasive sampling of biomarkers for diagnostic purposes available also outside highly specialized centers in the future.

### Correlation to clinical parameters

We found only weak associations between NFL in CSF and plasma and clinical symptoms and no association to outcome after shunt surgery, age or disease duration. A previous study including patients with idiopathic and secondary NPH found that increased NFL_CSF_ correlated with worse gait, balance and cognitive performance with stronger correlations for some of the clinical measures possibly related to the inclusion of secondary cases [16]. Recently we found weak correlations between higher CSF NFL and worse MMSE performances preoperatively as well as postoperatively [34]. These findings are replicated in this study, for the total iNPH scale score as well as for the subdomains of gait, balance, and neuropsychology. The neuropsychological tests included in the iNPH scale are selected to detect iNPH-specific cognitive decline, whereas MMSE is a rather crude measurement of cognitive function, probably more prone to capture also other causes of cognitive decline such as comorbid AD pathology or subcortical vascular dementia. We cannot rule out though, that in some iNPH patients such a comorbidity signals increased NFL concentrations and explain worse cognitive performance on the iNPH scale, a view supported by the similar correlations between MMSE and phosphorylated tau found in the same study [34]. However, correlations of similar strength also for gait and balance as well as for the total iNPH scale score lends support to the view that NFL elevation in some way, albeit weakly, is related to development of clinical symptoms in iNPH.

We found no association between NFL and age in patients with iNPH, in controls however, there was a weak correlation between NFL_plasma_ and age. These findings are consistent with other studies, where there seems to be a clearer age-related increase in NFL in healthy individuals, whereas there are more divergent results in cohorts with neurological disease [9]. In the aggregate, our study and previous reports indicate that NFL cannot be used as a sensitive marker of clinical symptoms, nor be used to predict outcome after shunting in iNPH.

### Changes induced by shunt surgery

In this study, NFL_CSF_ increased postoperatively while NFL_plasma_ did not. Studies describing postoperative changes of NFL_CSF_ have been contradictory and both increases [10, 14] decrements [16] and unchanged levels [11] in lumbar CSF in comparison to preoperative values have been reported. Diverging data has been attributed to different time span of postoperative sampling [35] and in a recent study of longitudinal changes, a temporary increase in CSF NFL induced by shunt surgery that was normalized after 6–9 months was found [36]. The postoperative sampling in our study was performed median 9 months postoperatively why our results contradict those of Lukkarinen et al. This discrepancy deserves further study.

### Strengths and limitations

We consider the sample of iNPH patients representative and comparable to earlier studies examining the concentration of NFL in CSF in patients with iNPH in comparison to controls. Patients were diagnosed according to diagnostic guidelines and well characterized regarding clinical symptoms and outcome. Controls were recruited from a population-based sample and matched for age and gender. Limitations include the lack of CSF in controls which limits the possibility to compare CSF concentrations of NFL between patients and healthy individuals, but this has been done in numerous previous studies [37, 38]. Further, we could not investigate the correlation between NFL in CSF and plasma in healthy controls, however, we find no reason to suspect that our healthy individuals should differ from those in other studies. Our sample of patients and controls was rather small, introducing a risk of type II error. We did not make any corrections for multiple comparisons to reduce risk of false negative findings and since we consider this study exploratory. The possibility that the shunt surgery might have some long-lasting effects on CSF and plasma NFL concentrations also call for additional follow-up samples in this cohort, e.g., 1- or 2-years post-surgery.

NFL_CSF_ was determined using a standardized ELISA and routine samples were used. Determining the concentration of NFL_CSF_ on the Simoa might increase the correlation [22]. On the other hand, the methodology used herein mirrors the standard clinical setting, where NFL_CSF_ is determined by ELISA and NFL_plasma_ by Simoa and makes it easier to transfer results to everyday routine. We used standard CSF samples in combination with stored plasma samples. Even though this could affect the results, NFL has shown high stability under different pre-analytical conditions, such as contamination, repeated freeze–thaw, delayed processing or long-term storing [39].

## Conclusions

Plasma NFL is increased in iNPH patients compared with healthy controls and concentrations correlate with CSF NFL, implying that plasma NFL can be used to assess evidence of axonal degeneration in iNPH. This finding supports the view that plasma NFL concentrations reflect CSF concentrations, which opens a window for plasma samples to be used in future studies of other biomarkers in iNPH. NFL is however probably not a clinically useful marker for diagnosis or for prediction of outcome in iNPH.

## Data Availability

Data will be made available upon reasonable request.
